# Influence of local industrial changes on reef coral calcification

**DOI:** 10.1038/s41598-020-64877-6

**Published:** 2020-05-12

**Authors:** Saori Ito, Tsuyoshi Watanabe, Megumi Yano, Takaaki K. Watanabe

**Affiliations:** 1 Department of Natural History Sciences, Faculty of Science, Hokkaido University, Sapporo, 060-0810 Japan; 2 KIKAI institute for coral reef sciences, Kikai town, 891-6151 Japan

**Keywords:** Marine biology, Geochemistry, Sustainability

## Abstract

Coral reefs are currently facing multiple disturbances caused by natural/anthropogenic factors. Recent industrial development might influence reef environments and ecosystems; however, few direct comparisons of coral calcification with the histories of local industries exist. We show the coral Ba/Ca record and growth histories for 46 years collected from Sumiyo Bay, Amami-Oshima Island, Japan. Coral Ba/Ca was mainly controlled by the sediment loads in seawater, which are introduced through the two local rivers. Coral Ba/Ca records have been characterized by two distinct historical periods: the decadal fluctuation corresponding to the traditional silk fabric industry (1960s ~ 1995) and the increasing trend corresponding to the development of quarries and the construction industry (1996 ~). Coral Ba/Ca records and local industrial histories were also linked to coral calcification. A long-term quantitative assessment of reef environments and local industrial changes could provide an evaluation of the survival strategies of reef-building corals in the future.

## Introduction

Healthy coral reefs supply a wide range of benefits to local communities around the world (e.g., an attraction for tourism, stable livelihoods in coastal areas, and a stable habitat for marine life). Coral reefs are currently facing multiple disturbances caused by natural/anthropogenic factors at a local and global scale (e.g., global warming, sea-level change, extreme meteorological phenomena, ocean acidification, land-/marine-based pollution, and sedimentation). Approximately 75% of the world’s coral reefs are rated as threatened when local threats are combined with thermal stress^1,2^. Coral reefs will expand their habitats to higher latitudes corresponding to global warming^3^. Annual mean precipitation in the high latitudes and the equatorial Pacific are likely to increase, and extreme precipitation events will very likely become more intense and more frequent, which would exacerbate sediment runoff to the coral reefs^1,4^. In addition, the intensity of human activities is expected to increase, leading to sediment and nutrient runoff, which can affect reef corals^1^. Corals are sensitive to the impacts of sedimentation and turbidity^5^. Decreased coral calcification causes a reduction or cessation of reef growth and may trigger a reduction in biodiversity on reefs^6–8^. Most of the recent reef assessments on the natural/anthropogenic sediment load were based on temporal and qualitative methods, for example, coral bleaching observations and reef cover monitoring^1,9,10^. However, temporal and qualitative methods cannot precisely demonstrate long-term changes in sediment load. Moreover, it was difficult to precisely assess the effects of the reef disturbance and coral calcification responses in the area where *in situ* data on rivers and estuaries (e.g., water quality, river discharge, and water temperature) are limited. There is no report directly comparing coral calcification with local industrial histories with multidecadal time series. Here, we show the quantitative relationship among local industrial histories, the changes in the reef environments as captured in the coral geochemistry record, and coral calcification responses in Sumiyo Bay, Amami-Oshima Island, Japan. Massive coral skeletons (e.g., *Porites* sp.) provide archives of various proxies for long-term environmental and climatic reconstructions with a monthly or biweekly resolution. The barium/calcium ratio (Ba/Ca) in the coral skeleton provides a long-term record of sediment load in seawater associated with precipitation (heavy rainfall events), river discharge (flood events), coastal development, and coastal land use^11–16^. Coral calcification could provide a relative scale of the impacts of reef disturbance, such as natural and anthropogenic sediment loads with a long time series. The Ba/Ca in the Sumiyo coral skeletons was mainly controlled by the amount of the desorbed Ba^2+^ from the sediment, which is transported from the two rivers (Sumiyo River and Yakugachi River) through mangrove forests. Coral Ba/Ca records can be characterized by two distinct periods corresponding to the thriving local industries. Our study inferred that natural and anthropogenic sediment loads would be one of the controlling factors of coral calcification.

## Study area

**Figure 1:**
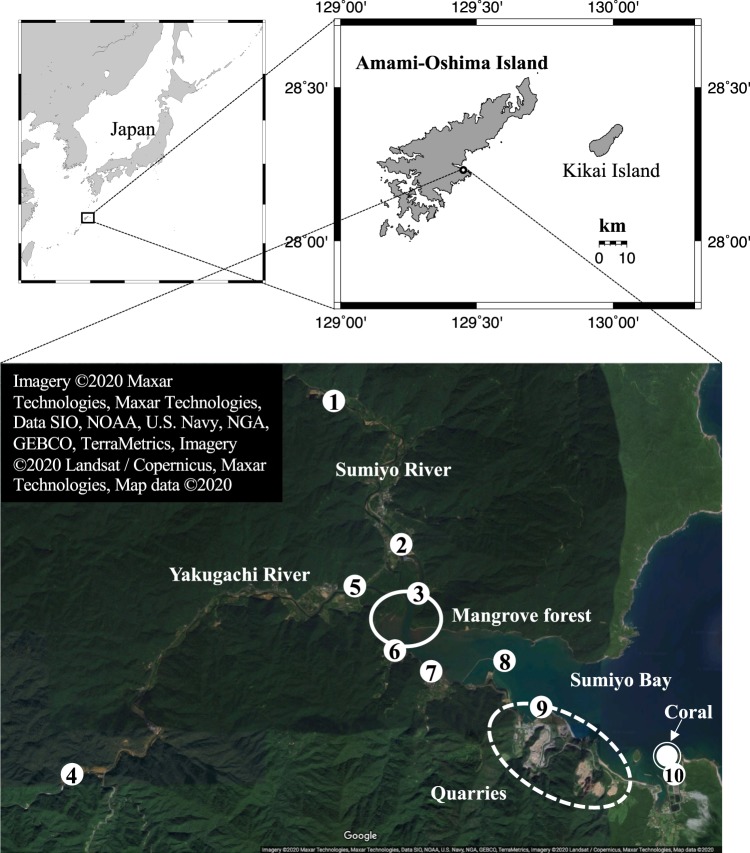
Regional map around the sample site (Sumiyo Bay, Amami-Oshima Island). Sumiyo River and Yakugachi River flow into Sumiyo Bay. The solid line area indicates a mangrove forest located at the rivers’ mouth, and the broken line area shows quarries in the southern coastal area of Sumiyo Bay. The white double-circle shows the site where *Porites* coral was drilled in this study. The distance between the rivers’ mouth and the sampling site was 4.5 km. The white single circles with a number show the site where the water samples were collected (No. 1, 2: Sumiyo River; No. 3: mangrove forest; No. 4, 5: Yakugachi River; No. 6, 7: Sumiyo Bay (brackish water); and No. 8, 9, 10: Sumiyo Bay (seawater)). The map figures (Japan, Amami-Oshima Island, and Kikai Island) were generated using Generic Mapping Tools (GMT v. 5.3.3)^59^. Satellite image (the Sumiyo area) was obtained by Google Maps (https://www.google.co.jp/maps/); Imagery ©2020 Maxar Technologies, Maxar Technologies, Data SIO, NOAA, U.S. Navy, NGA, GEBCO, TerraMetrics, Imagery ©2020 Landsat/Copernicus, Maxar Technologies, Map data ©2020. These figures were modified using Microsoft® PowerPoint for Mac (v. 16.35).

We drilled a coral core in Sumiyo Bay (Fig. 1), the eastern part of Amami-Oshima Island, Kagoshima Prefecture, Japan, in October 2014. Geologically, this area consists of sandstone, mudstone, and shale. The Sumiyo River (a 15.5 km flow path length and a 48.5 km^2^ basin area) and Yakugachi River (a 15.1 km flow path length and a 47.8 km^2^ basin area) run to Sumiyo Bay, and a mangrove forest covers the estuary. These rivers have recently caused river flooding four times (June 1977, September 1990, August 1991, and October 2010) over the past 46 years^17^. Despite such a situation, *in situ* data on rivers, estuaries, and bay (e.g., water quality, turbidity, salinity, river discharge, and water temperature) has not been monitored.

## Results and Discussions

### Ba/Ca profiles in the porites coral and Ba concentration in river- and sea-waters

**Figure 2:**
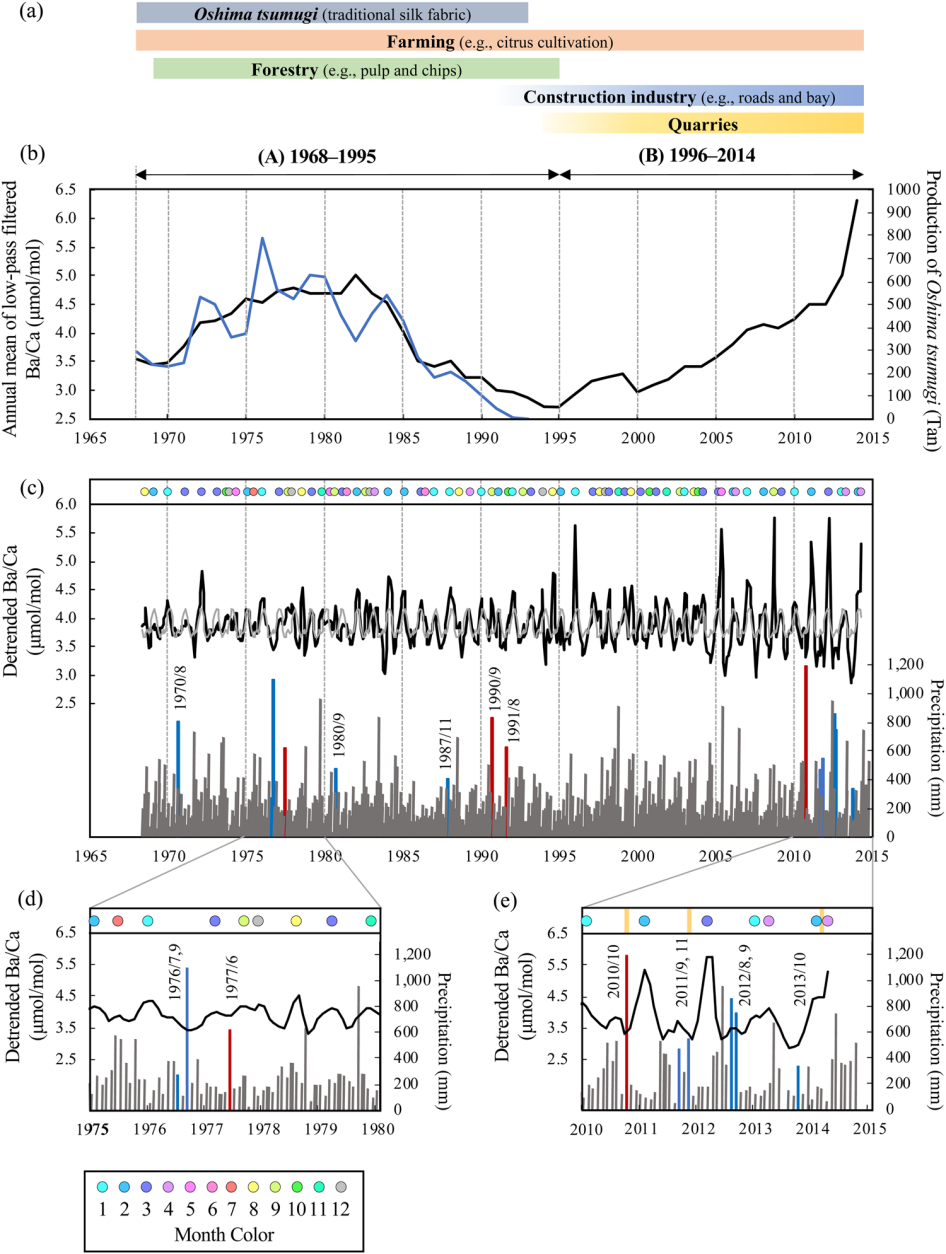
Time series of the skeletal Ba/Ca and land-use history (industrial development). **(a)** Land-use history (industrial development) in Sumiyo town from 1968 to 2014. In the A-period (1968~1995), *Oshima tsumugi* production and forestry prospered^17,28,29^. In the B-period (1996 ~ 2014), the construction industry and quarries prospered^17,28,29^. Farming also prospered during both periods^17,28,29^. **(b)** Annual mean of low-pass filtered skeletal Ba/Ca (in black) compared with the production of *Oshima tsumugi* (in blue). The low-pass filtered skeletal Ba/Ca was isolated by Gaussian bandpass filter (frequency = 0; bandwidth = 0.00137)^53^ from the raw skeletal Ba/Ca data, which is shown in Supplementary Fig. 2f (see also Materials and Methods). The standard unit of the production of *Oshima tsumugi* is “Tan” (in Japanese), which is defined as approximately twelve meters long and thirty-seven centimeters wide. **(c–e)** Detrended skeletal Ba/Ca compared with monthly precipitation. The detrended skeletal Ba/Ca was obtained by Gaussian bandpass filter (frequency = 0; bandwidth = 0.00137), as notch filtered data^53^. The gray line on the detrended skeletal Ba/Ca record indicates the mean seasonal cycles (MSC). The monthly color circles show the skeletal Ba/Ca high-peaks, which determined the difference between the mean value of MSC and the detrended data (see Methods and Materials). The blue and red precipitation areas show typhoons or heavy rainfall events (blue) and river flood disasters corresponding to typhoons or heavy rainfall (red)^17,30,31^. The month and year of heavy rainfall or river flooding events are shown as “YYYY/MM.” The yellow bars in (**e**) show the landslide events around the coastal area^30^.

The *Porites* coral specimen showed 46 clear annual density bands (Supplementary Fig. 1). We analyzed skeletal Sr/Ca and skeletal Ba/Ca records for 46 years from 1968 to 2014 (Supplementary Fig. 2a,f). The 46-year skeletal Ba/Ca record derived from the Sumiyo coral showed multidecadal fluctuations, which seemed to be larger than seasonal variability. Multidecadal fluctuation in the skeletal Ba/Ca record was isolated by Gaussian bandpass filter (i.e., low-pass filtered skeletal Ba/Ca; Fig. 2b, see also Materials and Methods). The detrended skeletal Ba/Ca showed spike-like patterns (Fig. 2c).

The coral skeletal Ba/Ca reflects seawater Ba concentration (Ba/Ca_coral_/Ba/Ca_seawater_ ≒ 1)^18^. Our data on the Ba concentration in seawater ranged from 32.1 to 45.1 nmol/kg (Supplementary Fig. 3). The mean value of measured Ba concentration in seawater (39.0 nmol/kg) mostly agreed with the variable range of the coral-derived Ba concentration in seawater in Sumiyo Bay (Supplementary Fig. 4), and it was slightly higher than the reported values in previous studies (Supplementary Table 1). There was a strong negative correlation between the Ba concentration in the water samples and the *in situ* salinity in the Sumiyo region (Supplementary Fig. 3, r = −0.89; *p* < 0.0001; *n* = 14). Ba is released/desorbed from particulate matter by ion exchange with seawater Mg^2+^ and Ca^2+^ ions in a low-salinity estuary^19,20^. The Ba concentration in seawater in Sumiyo Bay is controlled by the amount of the desorbed Ba^2+^ (in the estuary) from the Ba-containing sediment, which is transported from the two rivers through the mangrove forests with tides and precipitation effects. The primary source of Ba in the estuary is suspended fluvial sediment particles, which are transported by rivers towards the ocean with freshwater/flood plumes^19–21^. The river flood event would lead to an increase in the river end-member Ba concentration^21^. The desorbed Ba^2+^ from the particulate matter is readily substituted for Ca^2+^ in the coral aragonite lattice in proportion to the aqueous Ba/Ca ratio^18,22,23^.

### Multidecadal fluctuation in skeletal Ba/Ca

The trend of the low-pass filtered skeletal Ba/Ca was analyzed by SiZer trend analysis^24^ (see Materials and Methods). We found a trend shift point in 1995 ~ 1996, which is the significant increase trend changed into the significant decrease trend in <25 years of filter width (Supplementary Fig. 5). Thus, we divided the time series into two periods in this study: 1986 ~ 1995 (A-period) and 1996 ~ 2014 (B-period). Ba/Ca in corals might be controlled by following scenarios: (1) Sea surface temperature (SST) components of Ba/Ca^22,25^, (2) Ba uptake in sea surface due to upwelling^22,26,27^, and (3) Ba-containing sediments transported from the river (freshwater/flood plumes)^11–16^. For our skeletal geochemical record, the annual mean of low-pass filtered skeletal Ba/Ca was not correlated with low-pass filtered skeletal Sr/Ca (Supplementary Fig. 6a, r = −0.17; *p* = 0.26; *n* = 47), and it suggested that the skeletal Ba/Ca did not show similar variations with SST. Therefore, the skeletal Ba/Ca has not been affected by SST on the multidecadal scale. Moreover, the upwelling effect in this study was very small because the influence of the Ryukyu Current System was not remarkable and the correlation coefficient value (*r*) between the low-pass filtered skeletal Ba/Ca and the low-pass filtered coral derived δ^18^O_seawater_ was low (Supplementary Fig. 6b, r = 0.29; *p* < 0.05; *n* = 47, see also Supplementary text). Thus, there was the other control factor of the annual mean of low-pass filtered skeletal Ba/Ca in Sumiyo Bay, for example, natural/anthropogenic sediment load.

Ba/Ca in corals reflects anthropogenic sediment load in the coastal area (e.g., land-use, coastal development)^11,13,15^. The industrial development around Sumiyo Bay in the past 46 years^17,28,29^ can also be divided into two periods (A-period and B-period, Fig. 2a), and it was agreed with the significant trends shift in the low-pass filtered skeletal Ba/Ca. In the A-period, from the 1960s to 1995, the production of “*Oshima tsumugi*” and forestry prospered^17,28^. *Oshima tsumugi* is a traditional silk fabric on Amami-Oshima Island. It is made by “mud dyeing” with plant-derived tannic acid pigments and iron oxide contained in mud fields. In the A-period, there were many mud fields for dyeing along rivers, and the process of mud washing-off was carried out in the rivers^17^. Forestry in the A-period produced a substantial amount of pulp and chips (a timber factory in Sumiyo village closed in 1995)^17,28,29^. In the B-period, from 1996 to the present, the quarry and the construction industries (e.g., public works such as construction and maintenance work on roads, rivers, and the bay) prospered^17,28,29^. In addition, the farming (e.g., citrus cultivation) also prospered during both periods^17,28,29^. These local industries might have influenced the reef environment in Sumiyo Bay. It is necessary to compare the local archives on the change in the thriving industries with coral calcification responses to understand the anthropogenic reef disturbance.

**Figure 3:**
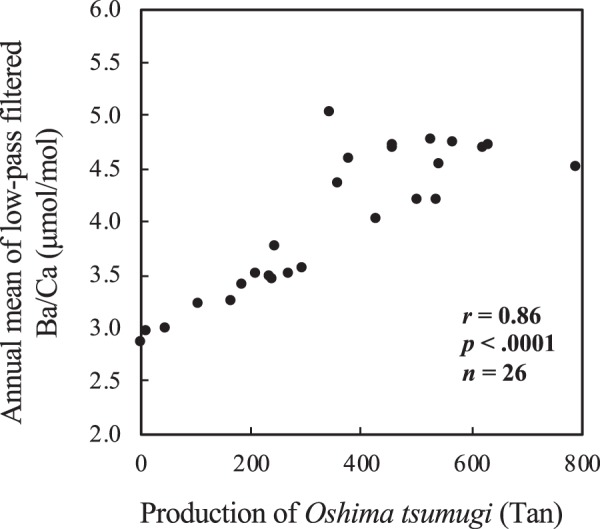
Relationship between annual mean of low-pass filtered skeletal Ba/Ca and the production of *Oshima tsumugi*. The time series of the annual mean of low-pass filtered skeletal Ba/Ca are shown in Fig. 2b. Note that the period of comparison was from 1968 to 1993.

The low-pass filtered skeletal Ba/Ca record and the annual production of *Oshima tsumugi* showed similar patterns (Fig. 2b, see also Materials and Methods). There was a significant positive relationship between the low-pass filtered skeletal Ba/Ca and the production volume of *Oshima tsumugi* (Fig. 3, *r* = 0.86; *p* < 0.0001; *n* = 26; from 1968 to 1993). This result suggests that the multidecadal fluctuation in the skeletal Ba/Ca in the A-period reflected the mud or sediment load in Sumiyo Bay due to “mud dyeing” (including the silk thread dyeing at the muddy fields, the mud washing-off process carried out in the rivers, and then the mud was transported to the ocean by the rivers). In addition, deforestation in the A-period also could have affected the low-pass filtered skeletal Ba/Ca. In the A-period, the sediment or mud may have constantly been flowing out into Sumiyo Bay due to forestry and *Oshima tsumugi* production. On the other hand, in the B-period, the low-pass filtered skeletal Ba/Ca had an upward pattern (Fig. 2b). The multidecadal fluctuation in the skeletal Ba/Ca in the B-period might have reflected an increase in sediment load due to rapid industrial development. In the B-period, a large amount of sediment flowed into Sumiyo Bay, which would be caused by the construction industry and quarries. In addition, the quarry collapse events (October 2011; November 2011; March 2014)^30^ would cause sediment input sporadically into Sumiyo Bay (Fig. 2e).

### Detrended skeletal Ba/Ca

The detrended skeletal Ba/Ca record was characterized by spike-like patterns (i.e., high-peaks). Skeletal Ba/Ca high-peaks were recorded 43 times in the A-period and 33 times in the B-period (Fig. 2c). For the river flooding disasters associated with typhoons or heavy rainfall events^17,30,31^, the skeletal Ba/Ca high-peaks were recorded in September 1977 (with a 3-month time lag from the event), September 1990 (without a time lag), October 1991 (with a 2-month time lag), and February 2011 (with a 4-month time lag). The high-peak values in response to these events in the B-period were larger than those in the A-period (Fig. 2d,e). It is possible that the response of the coral skeleton to these events has changed due to local industrial development, throughout the two periods.

There are two possible reasons for the time lag of several months that occurred between the river flooding events associated with heavy rainfall events and the peaks of the skeletal Ba/Ca: (1) a delay in the desorption of Ba^2+^ in the mangroves and estuary and (2) a decrease in or cessation of skeletal growth (for the 2010 event). (1) The estuarine Ba^2+^ desorption processes that affect the Ba concentration in seawater are known to be complex. Moyer *et al*.^32^ noted a time lag of several months between the peak of skeletal Ba/Ca and the peak of the river discharge, which was explained by the sporadic release of Ba from estuarine or floodplain-stored sediments. However, a large amount of sediment and freshwater will flow into the bay rapidly and suddenly when the limit of the mangrove trapping mechanism is exceeded due to heavy rainfall, river floods, and mangrove collapses. A large amount of sediment that flows out without being trapped by mangroves will be stored in the floodplains and estuaries, and then, the desorption of Ba^2+^ becomes sporadic and slow due to tidal mixing. As a result, the increase in the Ba concentration in Sumiyo Bay will be delayed. (2) For the heavy rainfall disaster that occurred in October 2010, the daily precipitation of that day (622.0 mm) was highest in the 46 years past. The 2010 heavy rainfall specifically triggered a river flood and the collapse of mangrove forests, which caused a large amount of sediment and freshwater input into Sumiyo Bay^30,31^. The skeletal Ba/Ca record tracked the changes in sediment load due to the 2010 disaster showing an increasing trend for 4 months, and a maximum value of skeletal Ba/Ca was 5.32 μmol/mol (1.4 times the mean value over all periods). Similarly, the skeletal Ba/Ca record showed a time-lag signal that rose over several months during the heavy rainfall event that occurred in September and November 2011. Clear low-density bands were observed in the X-radiographs from 2010 to 2012 when the coral experienced heavy rainfall, river floods, and landslide events (Supplementary Fig. 1). Anomalous high-/low-density bands are skeletal growth responses to environmental stressors, for example, thermal stress, sedimentation, or substantial precipitation^33^. In addition, the skeletal growth disturbance proxy, ΔSr/Ca in the Sumiyo coral, showed high-peaks after the 2010 event (Supplementary Fig. 2g). The skeletal Sr/Ca and Mg/Ca in the Sumiyo coral recorded a low sea surface temperature (SST, 17.6~18.6 °C derived by SST-Sr/Ca and SST-Mg/Ca regression; Supplementary Figs. 2a,b and 7, see also Supplementary text) after the 2010 river flood event. This scenario suggests that cold freshwater flowed into Sumiyo Bay from the Sumiyo and Yakugachi Rivers in the aftermath of the heavy rainfall event. The temporal heavy rainfall also probably influenced on corals. Fallon *et al*.^34^ explained that the time lag of the skeletal trace element record was caused by the temporal decrease in or cessation of skeletal growth during winters with lower SST than 18 °C. Thus, skeletal growth was temporarily inhibited by the 2010 river flood event, which might have created a time lag.

### Seasonal pattern in skeletal Ba/Ca

**Figure 4:**
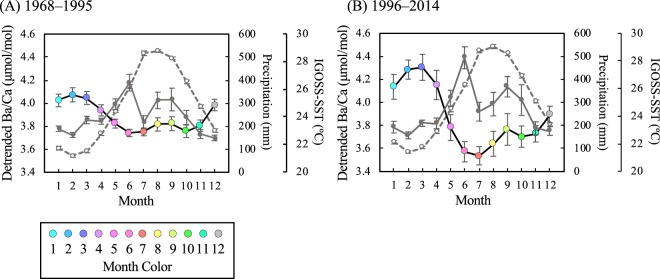
Seasonal pattern in the detrended skeletal Ba/Ca, precipitation, and SST in two periods. The black line with monthly color circles shows the seasonal pattern in detrended skeletal Ba/Ca (MSC, the gray line on Fig. 2c). The gray-solid line shows the seasonal variation in precipitation. The gray-dashed line shows the seasonal variation in IGOSS-SST^57^. Noted that the IGOSS-SST time series were from 1981 November to October 2014. The error bars show the standard error.

Figure 4 shows the annual detrended skeletal Ba/Ca record and the seasonal pattern in the two periods (A- and B-periods). The seasonal pattern in both periods was similar, while the variable ranges were different (A-period: 3.74 ~ 4.01 μmol/mol; B-period: 3.54 ~ 4.31 μmol/mol). The detrended skeletal Ba/Ca was significantly inversely correlated with precipitation and SST in both periods (A-period: Ba/Ca vs. precipitation: *r* = −0.59; *p* < 0.05; *n* = 12, Ba/Ca vs. SST: *r* = −0.87; *p* < 0.001; *n* = 12, B-period: Ba/Ca vs. precipitation: *r* = −0.64; *p* < 0.05; *n* = 12, Ba/Ca vs. SST: *r* = −0.88; *p* < 0.001; *n* = 12). There was no significant difference between the two periods for monthly precipitation and SST.

Only during the typhoon and heavy rainfall season (August to October) was the monthly mean of skeletal Ba/Ca showed a similar pattern with precipitation (A-period: *r* = 0.99; *p* < 0.05; *n* = 3, B-period: *r* = 0.96; *p* = 0.19; *n* = 3, Fig. 4). This result conflicts with the simple hypothesis that Ba-rich sediment particles are transported towards Sumiyo Bay by rivers with freshwater plumes associated with precipitation throughout the year. The estuaries serve as storage reservoirs for Ba-rich sediments^35–37^. The mangrove forest is one of the storage reservoirs. The mangrove can trap suspended sediments that are caused by river runoff or resuspension of bottom sediments^38–40^. The trapping mechanism has a limitation in which too much sedimentation can lead to mangrove mortality as the sediments asphyxiate the respiratory structures^41^. In addition, the mangroves are affected by both river (river runoff related to precipitation) and ocean effects (seawater inflow and outflow due to tide levels). Increased river flow due to precipitation makes sediment transport more active and leads to sediment inflow into mangroves^42^. Heavy rainfall and strong winds associated with typhoons or storms sometimes trigger river floods and landslides, which can cause the outflow of large amounts of sediments into the ocean with mangrove collapse^43^. Seawater flows into the mangroves during the springtide and flows out during the ebb tide^40,44,45^. Therefore, sediment transport is most active during springtide. The tide level in our study area was higher in summer and autumn (June to November) than in winter and spring (December to May). From August to October, typhoons and heavy rainfall events and river floods frequently occurred (Fig. 2c). Therefore, Ba-containing sediments might slowly flow into Sumiyo Bay from mangroves during winter to early summer (November to June, when there might be no influence of typhoons or heavy rainfall). On the other hand, the sediments exceeding the limit of the mangrove trapping mechanism might suddenly flow into Sumiyo Bay during summer to autumn (July to October, when typhoons, heavy rainfalls, and river floods occur frequently). From May to June, the sediment input to Sumiyo Bay is controlled by the mangrove trapping mechanism, although there is substantial precipitation. In addition, the low-pass filtered skeletal Ba/Ca (i.e., multidecadal fluctuation) did not show the SST effect; however, our skeletal Ba/Ca may have been influenced by SST on the seasonal scale. This finding is consistent with that of previous studies^22,25^. In particular, during winter to spring (December to May), the skeletal Ba/Ca may reflect the change in SST because the tide level is low and sediment transport from mangrove forests is considered to be less active. Thus, the skeletal Ba/Ca in the Sumiyo coral may reflect the mangrove system (affected by both tide level and precipitation) and SST variation on the seasonal scale. Moreover, the sensitivity (response) of the reef-building coral to sediment load has changed with the change in the rhythm of sediment outflow into Sumiyo Bay throughout the two periods. The results of the difference in the variable range of the seasonal trend between the two periods and the increase in spike-like signals in the B-period can be explained by the histories of the local industries.

### Coral calcification response to natural/anthropogenic sediment load

**Figure 5:**
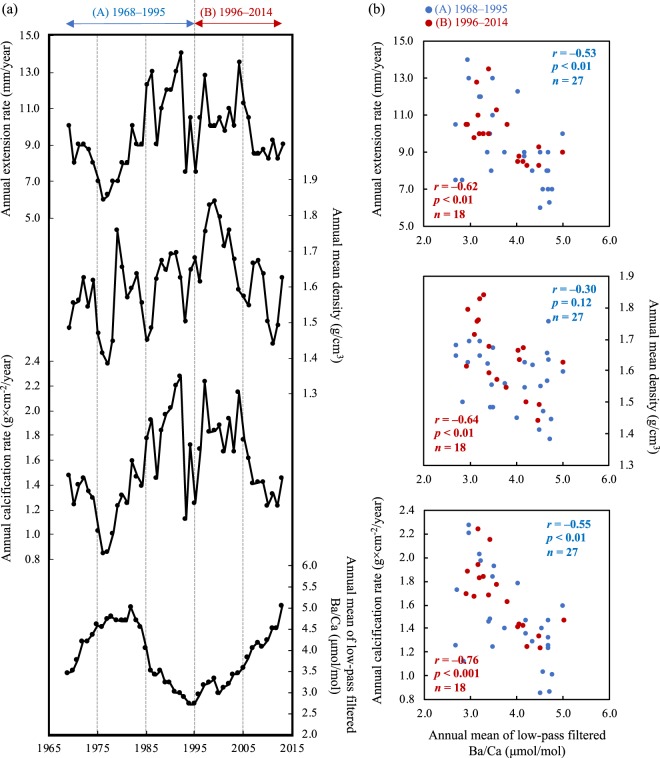
Skeletal growth parameters and correlation with annual mean of low-pass filtered skeletal Ba/Ca. **(a)** Time series of skeletal growth parameters and annual mean of low-pass filtered skeletal Ba/Ca. The annual skeletal calcification rate (g × cm^−2^/year) was obtained from the annual extension rate (mm/year) and annual mean density (g/cm^3^). The broken line on the age (year) separates two periods: (A) 1968 ~ 1995 and (B) 1996~2014. **(b**) Relationship between skeletal growth parameters and annual mean of low-pass filtered skeletal Ba/Ca in two periods: (A) and (B). The x-axis of all figures is the annual mean of low-pass filtered skeletal Ba/Ca.

Coral skeletal growth parameters (skeletal extension rate, skeletal density, and skeletal calcification rate) are obtained from annual bands and age models (see Materials and Methods). As shown in Fig. 5, there were the significant relationships between the annual mean of the low-pass filtered skeletal Ba/Ca and the annual extension rate in both periods (A-period: *r* = −0.53; *p* < 0.01; *n* = 27; B-period: *r* = −0.62; *p* < 0.01; *n* = 18). Besides, there were also the significant relationships between the low-pass filtered skeletal Ba/Ca and the annual calcification rate in both periods (A-period: *r* = −0.55; *p* < 0.01; *n* = 27; B-period: *r* = −0.76; *p* < 0.001; *n* = 18). Therefore, natural/anthropogenic sediment load was likely one of the controlling factors of coral calcification in Sumiyo Bay. Skeletal calcification is enhanced by light availability (i.e., inhabitation of light-enhanced calcification^46^). Numerous previous studies have reported that increasing the sediment load in seawater (i.e., high water turbidity) due to coastal runoff cause would reduce the coral light availability, and it will lead to disturbances in coral calcification^47–49^. Besides, the correlation coefficient *r*-value of skeletal Ba/Ca vs. annual calcification rate in the B-period (*r* = −0.76; slope: −0.36 ± 0.08) was larger than that in the A-period (*r* = −0.55; slope: −0.29 ± 0.09). There is a possibility that coral calcification in the B-period could more strongly respond to natural/anthropogenic sediment load than in the A-period.

## Conclusion

The skeletal Ba/Ca derived from the Sumiyo Bay coral was mainly controlled by the amount of the desorbed Ba^2+^ (in the estuary) from the Ba-containing sediment transported by the two rivers and mangrove forests. The tides and precipitation effects are shown as the seasonal pattern in skeletal Ba/Ca. Our skeletal Ba/Ca records were characterized by two distinct periods: (A) the multidecadal change with smaller seasonal variation during the 1960s to 1995 corresponding to the change in the traditional silk fabric industry and (B) the increase pattern with larger seasonal variability during 1996 to the present corresponding to the development of quarries and the construction industry. Thus, the sensitivity (response) of the reef-building coral to sediment load has changed with the change in the rhythm of sediment outflow into Sumiyo Bay throughout the two periods. Moreover, we concluded that natural (the sedimentation trapping mechanism in the mangroves, precipitation effects, and river flooding effects) and anthropogenic sediment loads (industrial development and land-use effect) were two of the controlling factors of coral calcification.

The coral growth record showed a response to coastal environmental change in the Sumiyo area. It indicates that Sumiyo corals can adapt to environmental changes (i.e., chronic sediment stress) if it is a gradual change over a long period (>approximately 50 years). The multiple and mixed components of natural and anthropogenic factors (i.e., human activity, climate change, and land-use change) will influence coral reefs^1,2,4,5,9,10^. A long-term quantitative assessment can simultaneously evaluate a reef environment and coral calcification. It can be essential to the discussion of survival strategies for reef-building corals in which precipitation and human activities will increase. If a sudden and larger environmental change than the natural/anthropogenic events reconstructed in this study occurs consecutively, it might have a stronger negative impact on the reefs. It is important to understand how natural/anthropogenic sediment loads lead to a reef disturbance and how corals respond to these loads to create a sustainable human society that can benefit from coral reefs. In addition, keeping the balance between natural/anthropogenic reef disturbance and the coral calcification response may also be needed to maintain reef health and reef biodiversity in the future. There are still many coastal areas where periodic monitoring surveys and *in situ* environmental data are not available. Massive coral skeletons (e.g., *Porites* sp.) can reconstruct reef disturbances with a high temporal resolution, and these skeletons will be a useful proxy for skeletal growth and calcification responses even in such coastal areas.

## Materials and Methods

### Coral core collection and geochemical analysis

We collected living *Porites* coral cores at a depth of 5.1 m inside Sumiyo Bay (28.23N 129.45E, Fig. 1) in October 2014. The distance between the mouths of the two rivers (Sumiyo River and Yakugachi River) and the coral sampling site was 4.5 km, and the distance from the nearest shore was 80 m. All cores were cut into 5 mm thick slabs. The slabs were rinsed with distilled water in an ultrasonic bath several times and dried at 50 °C for 24 h. X-ray photographs were taken at 50 kV and 1.0 mA for 8 seconds using a digital X-ray scanner (NAOMI, RF Co., Ltd, Japan) to observe annual density bands (Supplementary Fig. 1). Analytical lines were determined along the major growth axis. The slab edges formed into a ledge shape, 1.5 mm thick and 2.0 mm width along the analytical lines, using a micro drill. Skeletal powder samples for geochemical analysis were obtained by a precise microsampling method using a micro drill (1.8 mm diameter) and a PC controlled XY stage, as the method described in Watanabe *et al*.^50^. Microsampling intervals were 0.25 mm, and it was equivalent to 0.3 months resolution (the mean value of the annual skeletal extension rate was 9.6 mm/year). The milled area was cleaned by high-pressure air after each microsampling to avoid cross-contamination.

The skeletal Ba/Ca, Sr/Ca, and Mg/Ca analyses were performed by ICP-OES (iCAP 6200, Thermo Scientific, MA, USA) with an autosampler (ASX-260, Teledyne CETAC Technologies, NE, USA) and an ultrasonic nebulizer (U-5000AT, Teledyne CETAC Technologies). Powdered samples (115–128 μg) were dissolved in 0.57 ml of 25% high-purity HNO_3_ and then diluted with ultrapure water (Milli-Q), as described by Watanabe *et al*.^51^. We ultimately prepared sample solutions in which the Ca concentration was 9 ppm. Calibrations of the five gravimetric standard solutions yielded high correlation coefficients (*r*^2^) of 0.9999 for Ba, Sr, Mg, and Ca. To correct the instrumental drift, we measured the reference solution JCp-1^52^ at 5 sample intervals. The analytical uncertainties (1σ; relative standard deviations) were 2.24% or 0.18 μmol/mol for Ba/Ca, 0.17% or 0.01 mmol/mol for Sr/Ca, and 0.73% or 0.03 mol/mol for Mg/Ca. These profiles are shown in Supplementary Figs. 2a (Sr/Ca), 2b (Mg/Ca), and 2f (Ba/Ca).

The skeletal δ^18^O and δ^13^C analyses were performed using a mass spectrometer (Finnigan MAT 253, Thermo Scientific) coupled to a carbonate reaction device (Kiel II and IV Carbonate Device, Thermo Scientific). Powdered samples (100 ± 10 μg) were reacted with phosphoric acid at 70 °C in a carbonate reaction device. The CO_2_ gas produced in the reaction was then introduced into a coupled mass spectrometer. Skeletal δ^18^O and δ^13^C values are expressed in conventional delta notation in ‰ units relative to Vienna Peedee Belemnite (V-PDB) through measurements of the isotopic ratio of CO_2_ gas derived from Standards NBS-19 (δ^18^O = −2.20‰; δ^13^C = 1.95‰). The standard deviations (2σ; 95% confidence) were 0.02‰ and 0.03‰ for δ^18^O and δ^13^C, respectively. These profiles are shown in Supplementary Figs. 2c (δ^18^O) and 2d (δ^13^C).

### Statistical analysis

The skeletal Ba/Ca record was filtered with a Gaussian bandpass filter (frequency = 0; bandwidth = 0.00137, i.e., isolating >2 years or <2 years trend) using Analyseries 2.0.8^53^. The profiles of raw data and low-pass filtered data (isolating >2 years trend) are shown in Supplementary Fig. 2f. The detrended data (i.e., notch filtered data, isolating <2 years trend) are shown in Fig. 2c. The core-top data that showed anomalously high values (i.e., tissue layer, 4.1 mm = 5.1 months) were excluded from the filtering analysis. The high-peaks in the low-pass filtered skeletal Ba/Ca were determined the difference between the mean value of mean seasonal cycles (MSC) and the detrended data. When the peak points succeeded one another, the highest point was determined as a representative point (i.e., high-peak) in Fig. 2c–e.

Moreover, the time series of the low-pass filtered skeletal Ba/Ca was divided into two periods using SiZer (SIgnificant ZERo crossings of derivatives) method^24^. The analysis was performed using the statistical software R (v. 3.3.3)^54^ with Significant Zero Crossings for R package (v. 0.1–4)^55^. The package was obtained from https://CRAN.R-project.org/package=SiZer. As a result, there is a significant trend shift point in 1995~1996 (Supplementary Fig. 5). We divided the time series of the low-pass filtered skeletal Ba/Ca using the shift point (A-period: 1968–1995, B-period: 1996–2014). Because the increase and decrease trends were overlapped from 1975 to 1978 in a broad range of the filter width, we did not divide the time series of the low-pass filtered skeletal Ba/Ca.

### River- and sea-water sample collection and analysis

We collected 14 water samples in Sumiyo town (Fig. 1). Two of them were collected from the coral core drilling site (inside Sumiyo Bay) in October 2014. The other samples were collected from Sumiyo River, Yakugachi River, the mangrove forest, and Sumiyo Bay in July 2017. Water samples were filtered using a 0.45 μm Membrane filter (Merck Millipore, MA, USA), and it stored in PP bottles with 0.16 mL of 70% HNO_3_ (Trace Sure grade, FUJIFILM Wako Pure Chemical Corporation, Japan; pH = 2) to inhibit the change in trace elements caused by microorganisms. The bottles were stored refrigerated at 5 °C immediately upon collection. We measured *in situ* salinity when we collected the water samples.

The Ba contents in the water samples (both river- and sea-water) were analyzed using ICP-OES (iCAP 6200, Thermo Scientific) with a cyclonic spray chamber with a baffle. An analytical method for Ba in river- and sea-water was modified from Dehairs *et al*.^56^. We prepared four external standards in which the Ba and Sr solution standards (FUJIFILM Wako Pure Chemical Corporation) and 35 μg/L NaCl (Trace Sure grade, FUJIFILM Wako Pure Chemical Corporation) were mixed. The concentrations of Ba and Sr ranged from 3.0 to 30.0 ppb and 0.98 to 7.80 ppm, respectively. After four external standards analyses, we analyzed the water samples. To correct the instrumental drift, we measured a running standard (artificial seawater) at 5 sample intervals. The analytical precision of Ba in seawater was 1.185 (nmol/kg) based on a standard deviation of the running standard (1σ).

### Age determination and local archives

The age determination of the coral core was based on the relationship between skeletal Sr/Ca and monthly SST. The monthly IGOSS-SST time series at 1° × 1° (28.5N 129.5E) from November 1981 to October 2014 was derived from the Integrated Global Ocean Services System Products Bulletin (https://iridl.ldeo.columbia.edu/SOURCES/.IGOSS/.nmc/.Reyn_SmithOIv2/.monthly/.sst/)^57^. We used tie points to connect the values of skeletal Sr/Ca and SST record to establish each year, as follows; highest Sr/Ca and coolest SST (i.e., winter tied point) and lowest Sr/Ca and warmest SST (i.e., summer tied point). Time series were inserted by assuming constant growth rates during each of the tied points. The seasonal variation of SST dataset from November 1981 to October 2014 indicates that February had the lowest SST and August had the highest SST (Fig. 4). Therefore, the age model prior to November 1981 was also established using the tie points to connect the highest/lowest Sr/Ca and February/August.

Precipitation data were derived from the Japan Meteorological Agency with a monthly resolution (at Naze city, 28.380N 129.496E, from January 1968 to January 1998 and from December 1998 to December 1999) and Kagoshima Prefecture with a 10-minute resolution (at Sumiyo, 28.266N 129.408E, from February 1998 to November 1998 and from June 2000 to October 2014). The 10-minute time series were converted to a monthly resolution. In addition, monthly tide gauge data were derived from Permanent Service of Mean Sea Level (https://www.psmsl.org/) at the NASE station from 1968 to 2014.

Archived data on changes in land use and local industries, typhoon, heavy rainfall, landslide, and river flooding diseases in the past 46 years were based on literature surveys using “*Wakya sima nu ayumi*” (A history of Sumiyo, published in 2005, *in Japanese*)^17^, “*Amami Gunto no gaikyo*” (The overview of Amami Islands, an annual publication by Oshima Subprefecture in Kagoshima, Japan, *in Japanese*)^28^, national census data derived from Statistics Bureau of Japan (https://www.e-stat.go.jp/)^29^. Archived data on the 2010 heavy rainfall (river flooding) disaster was based on an academic research report published by Research and Education Center for Natural Hazards, Kagoshima University^30^ and an official report published by Amami City^31^. Interviews in Sumiyo town and local newspapers (*The Amami Shimbun* and *The Nankai Nichi-nichi Shimbun*) also provide us the supporting data. Data on the production of *Oshima Tsumugi* were provided by Oshima Subprefecture, in Kagoshima Prefecture. The information of the Sumiyo River and Yakugachi River (path length and basin area) was derived by the fundamental river management policy and river improvement plans established by Kagoshima Prefecture.

### Analysis of skeletal growth parameters

The extension rates of the corals were determined by measuring the distance between Sr/Ca maxima (SST minima). The skeletal density (g/cm^3^) was calculated using the CoreCal 2 program method^58^. The annual skeletal calcification rate (g × cm^−2^/year) was obtained from pairs of extension rates and annual skeletal density.

## Supplementary information

Supplementary Information.

## Data Availability

We plan to upload all data on the data repository at KIKAI Institute for coral reef sciences after acceptance.

## References

[1] Burke, L., Reytar, K., Spalding, M. & Perry, A. L. *Reefs at Risk Revisited*. (World Resources Institute, 2011).

[2] Hughes TP (2018). Spatial and temporal patterns of mass bleaching of corals in the Anthropocene. Science.

[3] Yamano H, Sugihara K, Nomura K (2011). Rapid poleward range expansion of tropical reef corals in response to rising sea surface temperatures. Geophys. Res. Lett..

[4] Pachauri, R. K. *Climate Change 2014: Synthesis Report. Contribution of Working Groups I. II and III to the Fifth Assessment Report of the Intergovernmental Panel on Climate Change* (IPCC, 2014).

[5] Erftemeijer PL, Riegl B, Hoeksema BW, Todd PA (2012). Environmental impacts of dredging and other sediment disturbances on corals: a review. Mar. Pollut. Bull..

[6] Brown BE, Howard LS (1985). Assessing the effects of ‘stress’ on reef corals. Advances Mar. Biol.

[7] Rogers CS (1990). Responses of coral reefs and reef organisms to sedimentation. Mar. Ecol. Prog. Ser..

[8] Fabricius KE (2005). Effects of terrestrial runoff on the ecology of corals and coral reefs: review and synthesis. Mar. Pollut. Bull..

[9] De’ath G, Fabricius KE, Sweatman H, Puotinen M (2012). The 27–year decline of coral cover on the Great Barrier Reef and its causes. Proc. Natl. Acad. Sci..

[10] Fisher R, Bessell-Browne P, Jones R (2019). Synergistic and antagonistic impacts of suspended sediments and thermal stress on corals. Nat. Commun..

[11] McCulloch M (2003). Coral record of increased sediment flux to the inner Great Barrier Reef since European settlement. Nature.

[12] Alibert C (2003). Source of trace element variability in Great Barrier Reef corals affected by the Burdekin flood plumes. Geochim. Cosmochim. Acta.

[13] Prouty NG, Field ME, Stock JD, Jupiter SD, McCulloch M (2010). Coral Ba/Ca records of sediment input to the fringing reef of the southshore of Moloka’i, Hawai’i over the last several decades. Mar. Pollut. Bull..

[14] Grove CA (2012). Spatial linkages between coral proxies of terrestrial runoff across a large embayment in Madagascar. Biogeosciences.

[15] Sowa K, Watanabe T, Kan H, Yamano H (2014). Influence of land development on Holocene *Porites* coral calcification at Nagura Bay, Ishigaki Island, Japan. Plos One.

[16] Saha N (2018). Seasonal to decadal scale influence of environmental drivers on Ba/Ca and Y/Ca in coral aragonite from the southern Great Barrier Reef. Sci. Total Environ..

[17] Sumiyo Village History Editorial Committee. *Wakya shima nu ayumi (a history of Sumiyo village*) Chapter 2, 107–315. (Sumiyo Village History Editorial Committee, 2005. *In Japanese*).

[18] Saha N, Webb GE, Zhao JX (2016). Coral skeletal geochemistry as a monitor of inshore water quality. Sci. Total Environ..

[19] Coffey M (1997). The behaviour of dissolved barium in estuaries. Estuar. Coast. Shelf Sci..

[20] Hanor JS, Chan LH (1977). Non-conservative behavior of barium during mixing of Mississippi River and Gulf of Mexico waters. Earth Planet. Sci. Lett..

[21] Sinclair DJ, McCulloch MT (2004). Corals record low mobile barium concentrations in the Burdekin River during the 1974 flood: evidence for limited Ba supply to rivers?. Palaeogeogr. Palaeoclimatol. Palaeoecol.

[22] Lea DW, Shen GT, Boyle EA (1989). Coralline barium records temporal variability in equatorial Pacific upwelling. Nature.

[23] LaVigne M, Grottoli AG, Palardy JE, Sherrell RM (2016). Multi-colony calibrations of coral Ba/Ca with a contemporaneous *in situ* seawater barium record. Geochim. Cosmochim. Acta.

[24] Chaudhuri P, Marron JS (1999). SiZer for exploration of structures in curves. J. Am. Stat. Assoc.

[25] Gaetani GA, Cohen AL, Wang Z, Crusius J (2011). Rayleigh-based, multi-element coral thermometry: A biomineralization approach to developing climate proxies. Geochim. Cosmochim. Acta.

[26] Shen GT (1992). Surface ocean variability at Galapagos from 1936–1982: calibration of geochemical tracers in corals. Paleoceanography.

[27] Tudhope AW, Lea DW, Shimmield GB, Chilcott CP, Head S (1996). Monsoon climate and Arabian Sea coastal upwelling recorded in massive corals from southern Oman. Palaios.

[28] Oshima Subprefecture (in Kagoshima Prefecture). *Amami Gunto no gaikyo* (The overview of Amami Islands), http://www.pref.kagoshima.jp/aa02/chiiki/oshima/chiiki/zeniki/gaikyou/index.html (2019).

[29] Statistics Bureau of Japan. National Census Data, https://www.e-stat.go.jp/ (2019).

[30] Kagoshima University. Reports on comprehensive scientific research for the 2010 heavy rainfall disaster in Amami (Research and Education Center for Natural Hazards, Kagoshima University, 2012. *In Japanese*).

[31] Amami City. Official report on the October 2010 heavy rainfall disaster in Amami. (Amami City, 2013. *In Japanese*).

[32] Moyer RP, Grottoli AG, Olesik JW (2012). A multiproxy record of terrestrial inputs to the coastal ocean using minor and trace elements (Ba/Ca, Mn/Ca, Y/Ca) and carbon isotopes (δ^13^C, Δ^14^C) in a nearshore coral from Puerto Rico. Paleoceanography.

[33] Lough JM, Cooper TF (2011). New insights from coral growth band studies in an era of rapid environmental change. Earth. Sci. Rev.

[34] Fallon SJ, McCulloch MT, van Woesik R, Sinclair DJ (1999). Corals at their latitudinal limits: laser ablation trace element systematics in Porites from Shirigai Bay, Japan. Earth Planet. Sci. Lett..

[35] Edmond JM, Boyle ED, Drummond D, Grant B, Mislick T (1978). Desorption of barium in the plume of the Zaire (Congo) River. J. Sea Res..

[36] Li YH, Chan LH (1979). Desorption of Ba and ^226^Ra from river-borne sediments in the Hudson estuary. Earth Planet. Sci. Lett..

[37] Dorval E, Jones CM, Hannigan R (2005). Chemistry of surface waters: Distinguishing fine‐scale differences in sea grass habitats of Chesapeake Bay. Limnol. Oceanogr..

[38] Wolanski, E., Mazda, Y. & Ridd, P. Mangrove hydrodynamics in *Tropical mangrove ecosystems* (eds. Robertson, A. & Alongi, D.) 43–46 (American Geophysical Union, 1992).

[39] Wolanski E (1995). Transport of sediment in mangrove swamps. Hydrobiologia.

[40] Furukawa K, Wolanski E, Mueller H (1997). Currents and sediment transport in mangrove forests. Estuar. Coast. Shelf Sci..

[41] Ellison J (1993). Mangrove retreat with rising sea level, Bermunda. Estuar. Coast. Shelf Sci..

[42] Sidik F, Neil D, Lovelock CE (2016). Effect of high sedimentation rates on surface sediment dynamics and mangrove growth in the Porong River, Indonesia. Mar. Pollut. Bull..

[43] Gilman EL, Ellison J, Duke NC, Field C (2008). Threats to mangroves from climate change and adaptation options: a review. Aquatic botany.

[44] Mazda Y, Sato Y, Sawamoto S, Yokochi H, Wolanski E (1990). Links between physical, chemical and biological processes in Bashita-minato, a mangrove swamp in Japan. Estuar. Coast. Shelf Sci..

[45] Mazda Y, Kobashi D, Okada S (2005). Tidal-scale hydrodynamics within mangrove swamps. Wetl. Ecol. Manag.

[46] Barnes, D. J. & Chalker, B. E. Calcification and photosynthesis in reef-building corals and algae in *Coral Reefs* (ed. Dubinsky, Z.) 109–131 (Elsevier, 1990).

[47] Hudson JH (1981). Growth rates in *Montastraea annularis*: a record of environmental change in Key Largo Coral Reef Marine Sanctuary, Florida. Bull. Mar. Sci..

[48] Dodge RE, Lang JC (1983). Environmental correlates of hermatypic coral (*Montastrea annularis*) growth on the East Flower Gardens Bank, northwest Gulf of Mexico. Limnol. Oceanogr..

[49] Crabbe MJC, Smith DJ (2005). Sediment impacts on growth rates of *Acropora* and *Porites* corals from fringing reefs of Sulawesi, Indonesia. Coral Reefs.

[50] Watanabe, T. *et al*. Coral sclerochronology: similarities and differences in coral isotopic signatures between mesophotic and shallow-water reefs in *Mesophotic Coral Ecosystems* (eds. Loya, Y., Puglise, K. & Bridge, T.) 667–681 (Springer, Cham, 2019).

[51] Watanabe T, Minagawa M, Oba T, Winter A (2001). Pretreatment of coral aragonite for Mg and Sr analysis: Implications for coral thermometers. Geochem. J..

[52] Okai. T, Suzuki A, Kawahata H, Terashima S, Imai N (2002). Preparation of a new geological survey of Japan geochemical reference material: Coral JCp-1. Geostand. Newsl.

[53] Paillard D, Labeyrie L, Yiou P (1996). Macintosh program performs time‐series analysis. Eos (Washington DC).

[54] R Core Team. R: A language and environment for statistical computing. R Foundation for Statistical Computing, Vienna, Austria, https://www.R-project.org/ (2018).

[55] Sonderegger, D. SiZer: SiZer: Significant Zero Crossings. R package version 0.1–4, https://CRAN.R-project.org/package=SiZer (2012).

[56] Dehairs F, Neybergh H, Hoenig M (1989). Direct determination of dissolved barium in sea water by inductively coupled plasma/atomic emission spectrometry. Anal. Chim. Acta.

[57] Reynolds RW, Rayner NA, Smith TM, Stokes DC, Wang W (2002). An Improved *In Situ* and Satellite SST Analysis for Climate. J. Climate.

[58] Sowa K, Watanabe T, Nakamura T, Sakai S, Sakamoto T (2013). Estimation of uncertainty for massive *Porites* coral skeletal density. JAMSTEC Report of Research and Development.

[59] Wessel P, Smith WH, Scharroo R, Luis J, Wobbe F (2013). Generic mapping tools: improved version released. Eos, Transactions AGU.

