# Supplementary material for: Influence of local industrial changes on reef coral calcification
Source: Sci Rep. 2020 May 12;10:7892. doi: 10.1038/s41598-020-64877-6 (PMC7217905; doi:10.1038/s41598-020-64877-6)
Supplement: Supplementary file 1 — Supplementary Information. [file 41598_2020_64877_MOESM1_ESM.pdf]

Supplementary Information

Influence of local industrial changes on reef coral calcification

Saori Ito [a], Tsuyoshi Watanabe [a, b, *], Megumi Yano [a], Takaaki K. Watanabe [a]

[a] Department of Natural History Sciences, Faculty of Science, Hokkaido University, Sapporo, 060-0810, Japan

[b] KIKAI institute for coral reef sciences, Kikai town, 891-6151, Japan

[*]Corresponding author: nabe@sci.hokudai.ac.jp (T. Watanabe)

Summary of this supplementary file:

Supplementary Figures 1 to 7

Supplementary Table 1

Response of the heavy rainfall, typhoon, and river flooding events on the coral skeletal proxies (Sr/Ca, and Mg/Ca, $\delta^{18}O$, and $\delta^{13}C$) and coral derived $\delta^{18}O_{seawater}$

Upwelling effect on the Sumiyo coral records

The notification of the skeletal Ba/Ca for detecting the sediment load

References

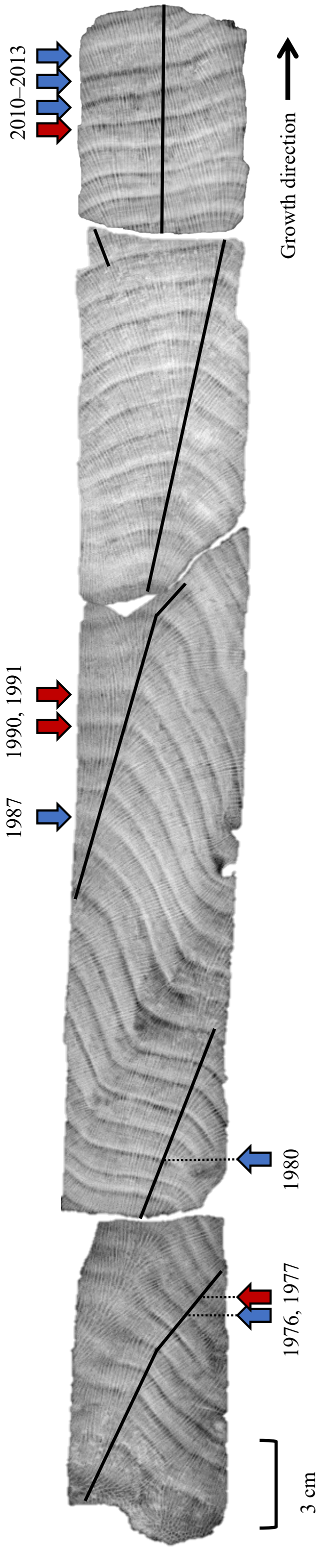

Supplementary Fig. 1. Images of X-radiographs (positive)

The black lines show analytical lines. The color arrows indicate the annual bands when the typhoon (blue; 1976, 1980, 1987, 2011, 2012, 2013) and river flood disasters (red; 1977, 1990, 1991, 2010) occurred[1-3].

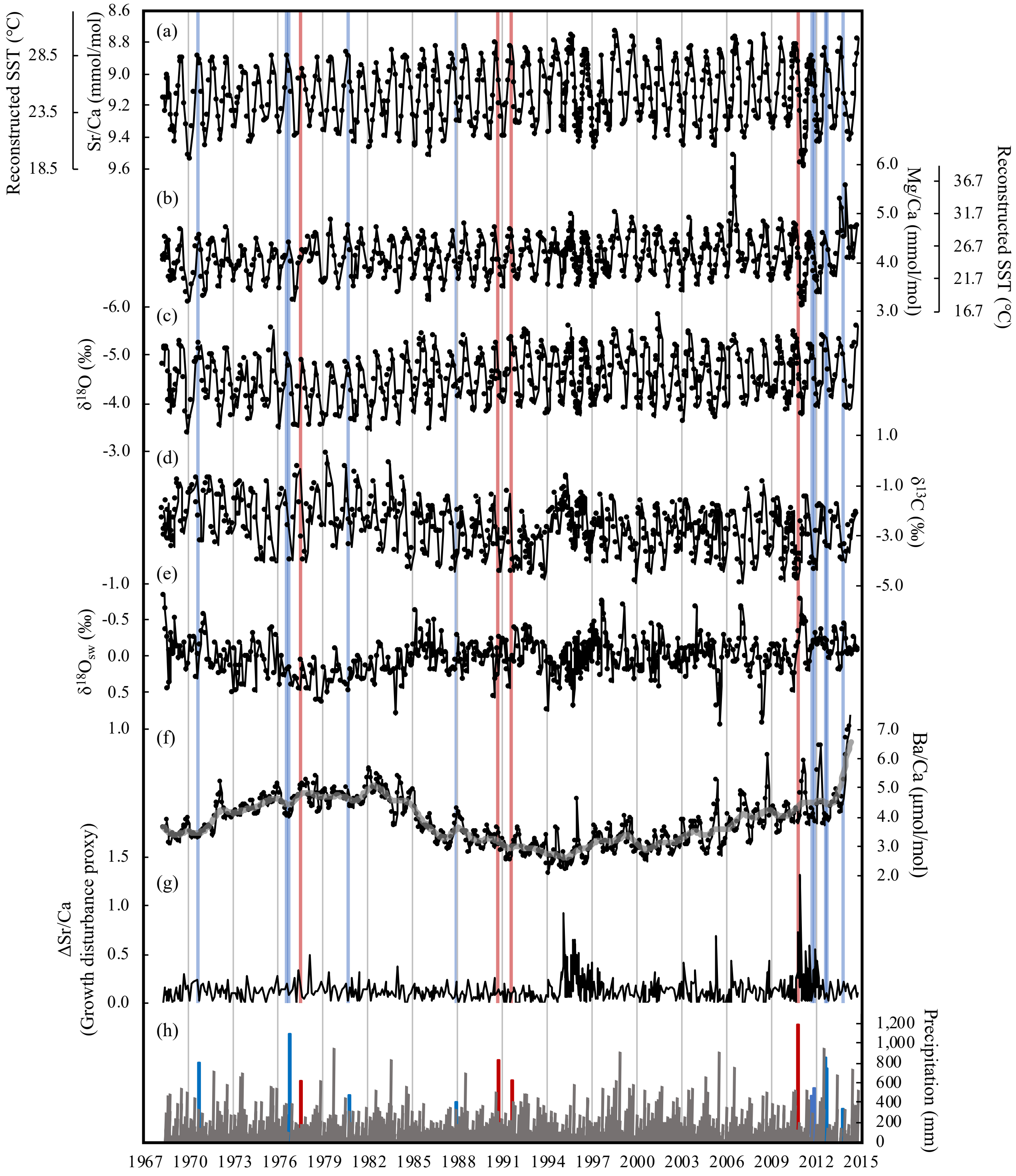

Supplementary Fig. 2. Coral-derived geochemical records and precipitation data back to 1968.

(a) Sr/Ca, (b) Mg/Ca, (c) $\delta^{18}O$, (d) $\delta^{13}C$, and (f) Ba/Ca are skeletal geochemical records. The y-axis on the skeletal Sr/Ca and Mg/Ca indicates measured data (mmol/mol) and reconstructed SST (°C). Both Sr/Ca-SST and Mg/Ca-SST regression are shown in Supplementary Fig. 7. The gray line on the skeletal Ba/Ca indicates low-

pass filtered data. (e) $\delta^{18}O$ of seawater ($\delta^{18}O_{sw}$) was calculated by skeletal Sr/Ca and $\delta^{18}O$[4-5]. The data was resampled with monthly resolution. (g) ΔSr/Ca shows the difference in the neighboring values in the skeletal Sr/Ca record. The coral skeleton could not record SST when skeletal growth was disturbed; therefore, a high value in ΔSr/Ca indicates a temporary growth disturbance. (h) The resolution of the precipitation is monthly. The blue and red areas of precipitation show typhoons or heavy rainfall (blue) and river flooding disasters, corresponding to typhoons or heavy rainfall events (red)[1-3].

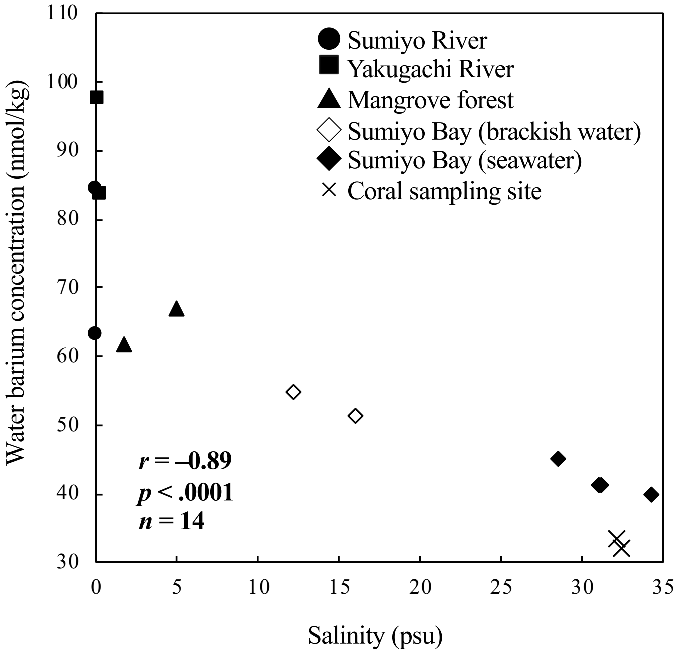

Supplementary Fig. 3. Correlation between the water barium concentration and the salinity in the Sumiyo area.

We collected 14 water samples from Sumiyo, Amami-Oshima Island (see Fig. 1). Two of them were collected from the coral drilling site in October 2014. The other samples were collected from the Sumiyo area (Sumiyo River, Yakugachi River, mangrove forest, and Sumiyo Bay) in July 2017. Salinity (in psu) was *in situ* data. Our water samples and salinity data were not affected by any typhoon, heavy rainfall, river flood, and landslide event.

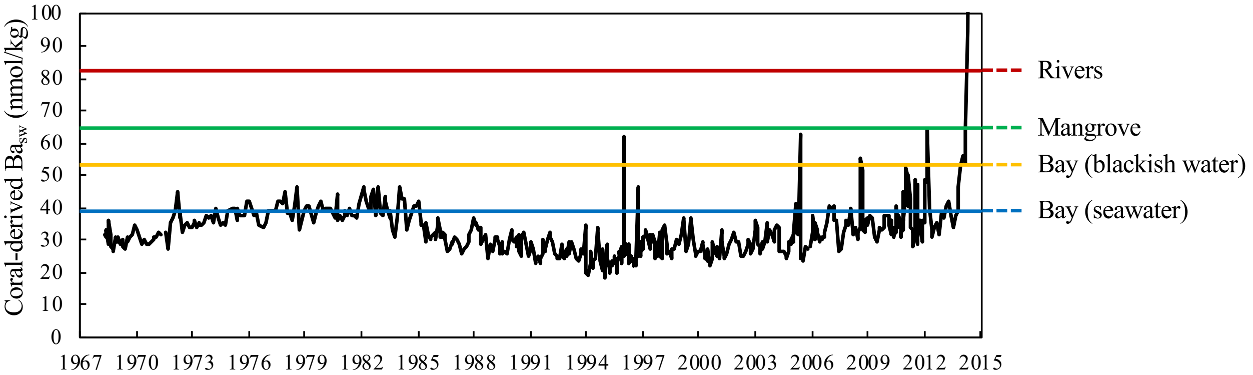

Supplementary Fig. 4. Variable range in measured Ba concentration in water samples and coral-derived Ba$_{sw}$

The color lines show the mean value of measured Ba concentration in the river- and sea-water samples (the raw data is shown in Supplementary Fig. 3). Coral-derived Ba concentration in seawater (Coral-derived Ba$_{sw}$) is calculated using a reporting regression for *Porites* sp. (LaVigne *et al.*[6], equation (2)).

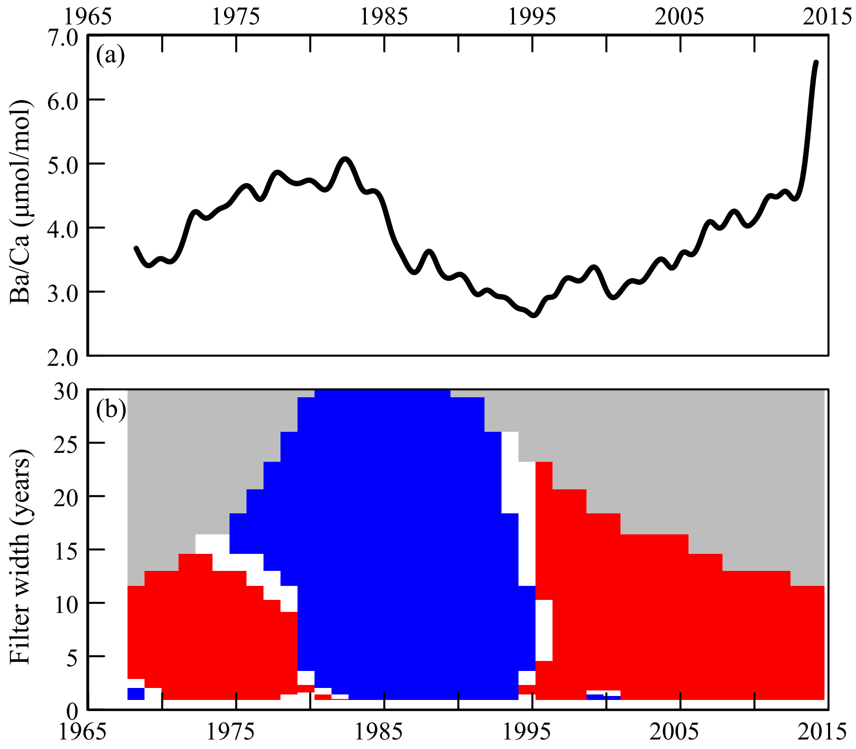

Supplementary Fig. 5. SiZer trend map for the skeletal Ba/Ca record

(a) Low-pass filtered skeletal Ba/Ca. This time series is the same as Supplementary Fig. 2-f (in gray line). (b) SiZer trend map for the low-pass filtered skeletal Ba/Ca[7-9]. A SiZer map is a way of examining when the p-th derivative of a scatterplot-smoother is significantly negative, possibly zero or significantly positive across a range of smoothing bandwidths[9]. The y-axis indicates the bandwidth values for which a smoothing function was calculated. Significant ($p < 0.05$) trends are shown as blue (significant decreasing) and red (significant increasing). The white color shows insignificant trends ($p > 0.05$). The gray color indicates the range that non-enough data in the filter width.

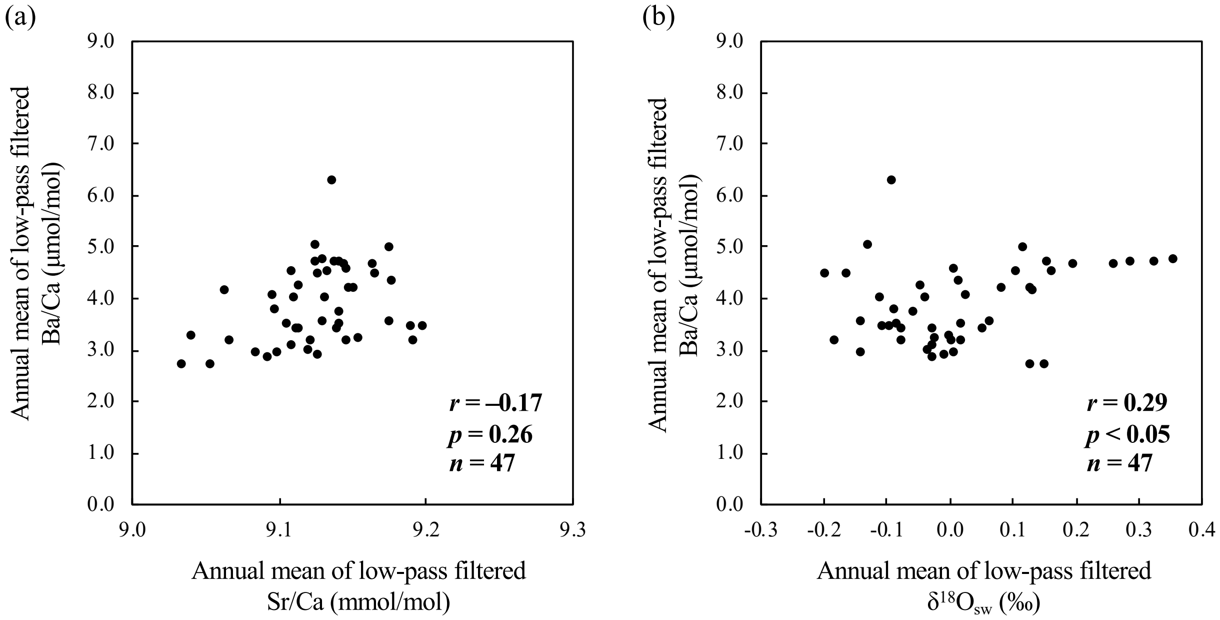

Supplementary Fig. 6. Correlation among low-pass filtered Ba/Ca, Sr/Ca, and coral derived $\delta^{18}O_{seawater}$

The time series of the annual mean of low-pass filtered skeletal Ba/Ca are shown in Fig. 2-b. Methodology for low-pass filtering for skeletal Sr/Ca and coral derived $\delta^{18}O_{seawater}$ was the same as skeletal Ba/Ca (see Materials and Methods in the text).

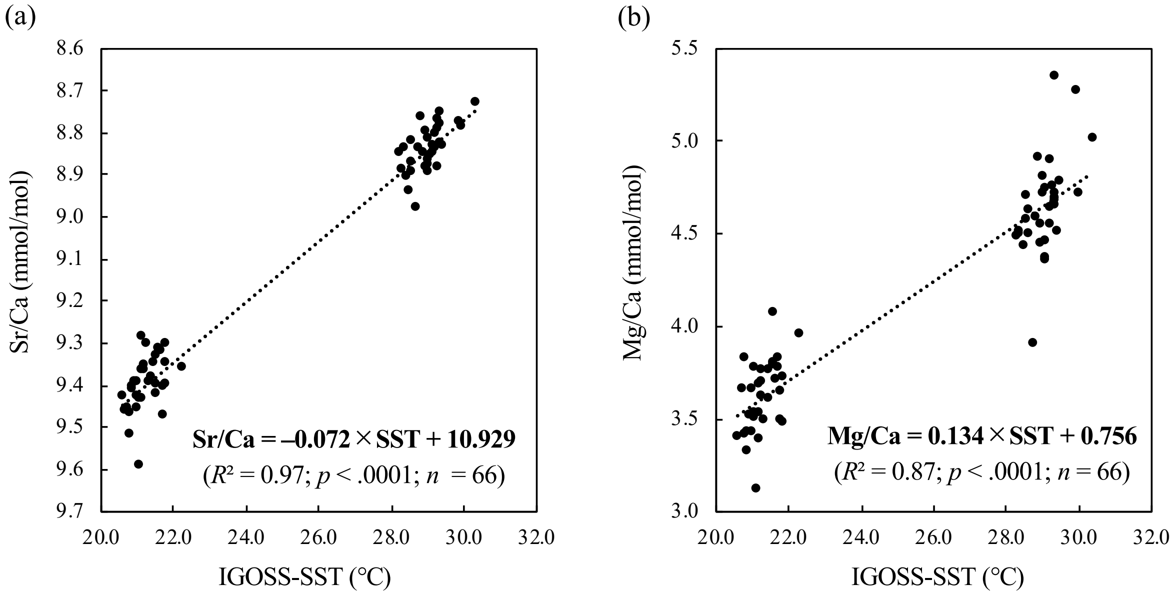

Supplementary Fig. 7. Coral Sr/Ca-SST and Mg/Ca-SST calibration for Sumiyo Bay, Amami Island

The dashed lines in both figure (a) and (b) show the mean calibration for each dataset with the calibration equations shown. The monthly IGOSS-SST time series was derived from the Integrated Global Ocean Services System Products Bulletin (https://iridl.ldeo.columbia.edu/SOURCES/.IGOSS/.nmc/.Reyn_SmithOIv2/.monthly/.sst/; see also Materials and Methods in the text). Obtained regressions are the following:

$$\text{Sr/Ca (mmol/mol)} = -0.072 \pm 0.002 \times \text{SST (°C)} + 10.929 \pm 0.041 \quad (1)$$

$$\text{Mg/Ca (mmol/mol)} = 0.134 \pm 0.007 \times \text{SST (°C)} + 0.756 \pm 0.167 \quad (2)$$

The slope of Me/Ca-SST regression for Sumiyo Bay, Amami-Oshima is –0.072 and 0.134 for Sr/Ca-SST and Mg/Ca, respectively. These results mostly agree with the previously published *Porites* Sr/Ca-[10] and Mg/Ca-SST[11] calibrations.

Site	Ba concentration in seawater (nmol/kg)	Sample	Reference
Lanyu Islet, southern Taiwan	33.4 - 32.8	Seawater	Yu *et al*. (2015)
Northeastern tropical Pacific	~ 35 - 42	Seawater	Esser & Volpe (2002)
Shirigai Bay, Japan	31 - 41	Estimated from skeletal Ba/Ca record	Fallon *et al*. (1999)
Ishigaki Island, Japan	26.1 - 32.0	Estimated from skeletal Ba/Ca record	Sowa *et al*. (2014)
Sumiyo Bay, Japan	32.1 - 45.1	Seawater	This study

Supplementary Table 1 **Reported data of the Ba concentration in sea water**

The reported data were derived from four sites: Lanyu Islet[12]; northeastern tropical Pacific that is less affected by upwelling[13]; Shirigai Bay, Kochi Prefecture, Japan[14]; and Ishigaki Island, Okinawa Prefecture, Japan[15]. Our data on the Ba concentration in seawater ranged from 32.1 to 45.1 nmol/kg, which seemed to be slightly higher than reported values in previous studies.

Response of the coral skeletal proxies (Sr/Ca, and Mg/Ca, $\delta^{18}O$, and $\delta^{13}C$) and coral derived $\delta^{18}O_{seawater}$ to the heavy rainfall, typhoon, and river flooding events

1) SST proxy

Skeletal strontium/calcium ratios (Sr/Ca)[16-18] and magnesium/calcium ratios (Mg/Ca)[11,19-20] are useful proxies for paleo-sea surface temperature (SST) variations. The skeletal Sr/Ca and Mg/Ca records showed a low-SST peak after the 2010 river flooding disaster (Supplementary Figs. 2-a and 2-b, see also text). It suggests that cold freshwater flowed into Sumiyo Bay from the two rivers (Sumiyo River and Yakugachi River) during the 2010 river flooding disaster. In the other events, the skeletal Sr/Ca and Mg/Ca did not show anomalous signals.

Coral skeletal oxygen isotope ratios ($\delta^{18}O$) reflect the SST and the oxygen isotope ratios in seawater related to the sea surface salinity[21-23]. The skeletal $\delta^{18}O$ record did not show the change in the habitat environments during the heavy rainfall, typhoon, and river flooding events (Supplementary Fig. 2-c). It may be caused by the complexity of controlling factors. The component of sea surface salinity would be masked by the component of SST in the skeletal $\delta^{18}O$ record.

2) $\delta^{18}O_{seawater}$ ($\delta^{18}O_{sw}$)

$\delta^{18}O_{sw}$ is available by combination skeletal Sr/Ca with skeletal $\delta^{18}O$, and it reflects sea surface salinity and evaporation[4-5,24-25]. The coral derived $\delta^{18}O_{sw}$ records showed a negative peak after the 2010 river flooding disaster (Supplementary Fig. 2-e). Similar to the low-SST signals in the skeletal Sr/Ca and Mg/Ca, it suggests that freshwater flowed into Sumiyo Bay from the two rivers (Sumiyo River and Yakugachi River) during the 2010 river flooding disaster. In addition, the temporal heavy rainfall would also affect the coral derived $\delta^{18}O_{sw}$. On the other hand, in the other events, the coral derived $\delta^{18}O_{sw}$ did not show anomalous signals. The difference might be caused by the daily precipitation during the event (the 1977 event: 281.0 mm; the 1990 event: 424.0 mm; the 1991 event: 325.5 mm; the 2010 event: 622.0 mm) or the impact of the river flooding (the 2010 river flooding event caused the most devastating

damage in the Sumiyo area in 46 years[1-3]). The negative peak in coral derived $\delta^{18}O_{sw}$ may be related to the magnitude (impact) of the river flooding and the amount of precipitation.

3) Coral light availability proxy

Coral skeletal carbon isotope ratios ($\delta^{13}C$) is mainly controlled by photosynthesis by symbiotic algae (zooxanthellae) which has a strong relationship to changes in water quality (coral light availability) and water depth[22,26-29]. It is a possibility that the skeletal $\delta^{13}C$ could reflect lower light availability (i.e., high turbidity) due to the increased sediment load corresponding to the heavy rainfall, typhoon, and river flooding events; however, the skeletal $\delta^{13}C$ record did not show any signal during these events (Supplementary Fig. 2-d). The complex in controlling factors of coral skeletal $\delta^{13}C$ has been reported by numerous previous studies, for example, the $\delta^{13}C$ of dissolved inorganic carbon in seawater[27,30-31], coral mass spawning[23], heterotrophic feeding[27,32], and respiration[27,33-35]. These factors might affect the skeletal $\delta^{13}C$ record in the Sumiyo coral, and they would mask the influence of temporal high turbidity due to the events.

Upwelling effect on the Sumiyo coral records

Several previous studies have reported that the skeletal Ba/Ca record could be affected by nutrient-rich upwelling events[36-38]. In general, upwelling events will lead to cold and eutrophic seawater from deep-sea due to the vertical water mixing. Vertical distributions of Ba in seawater are similar to those of nutrients. In the surrounding area of our study site (eastern part of Amami-Oshima Island; 28.23 N 129.45 E), the upwelling is only known on the shelf off northeast Taiwan[39-40] (25.68 N, 122.18 E). There has been no previous report that shows the evidence of the upwelling in the eastern Amami Islands and the upwelling effect on coral geochemical proxy collected in the Amami Islands[40]. Besides, there is the Ryukyu Current System, which has a northeastward current southeast of the Ryukyu Islands[41-43]. According to Thoppil *et al.*[43], the Ryukyu Current System in the southeast of Amami-Oshima Island (27-

28.5 N, 129.5-131.5 E) shows the significant strong patterns in 1998, 1999, and 2011. IGOSS-SST time series and skeletal Sr/Ca and Mg/Ca record (SST proxy, Supplementary Fig. 2-a) did not show the anomalous low-SST. There was no significant relationship between the low-pass filtered skeletal Ba/Ca and the low-pass filtered skeletal Sr/Ca (Supplementary Fig. 6-a, r = –0.17; p = 0.26; n = 47). It suggested that the skeletal Ba/Ca did not show similar variations with SST on the multidecadal scale. The Ryukyu Current System is weaker, deeper, and relatively unstable than the Kuroshio Current[43]. Therefore, the influence of the Ryukyu Current System to the coral proxies collected from Sumiyo Bay would be negligible. Moreover, a publishing data of seawater salinity variation showed a declining pattern with water depth, measured at 28 N 135.9 E[44] (the nearest station of this study area). The correlation coefficient value (r) between the low-pass filtered skeletal Ba/Ca and coral derived $\delta^{18}O_{seawater}$ was low (Supplementary Fig. 6-b, r = 0.29; $p < 0.05$; n = 47). Thus, we concluded that the upwelling effect in our skeletal Ba/Ca record would be very small.

The notification of the skeletal Ba/Ca for detecting the sediment load.

Skeletal Ba/Ca records the signals of heavy rainfall, river flooding, and sediment outflow into the ocean, as shown in this study and in previous studies. However, there are still unknown aspects of using skeletal Ba/Ca as a proxy. For example, our skeletal Ba/Ca record showed some peaks that did not coincide with the typhoons, heavy rainfall events, storms, landslides, or river flood events (e.g., January 1996, May 2005, and September 2008, see Fig.1-c). The peak in January 1996 may reflect the October 1995 tsunami events corresponding to the M_w 6.9 and 6.7 earthquakes. Irregular skeletal Ba/Ca peaks in coastal corals have been reported by previous studies[45-47]. A few previous studies have proposed various mechanisms for the Ba biogeochemistry that could explain the irregular skeletal Ba/Ca peaks[45,48-49]; however, these studies could not explain the whole biogeochemistry mechanisms in the skeletal Ba/Ca. Additional research is needed to understand in detail the biogeochemistry mechanisms in skeletal Ba/Ca, and this information will allow us to reconstruct reef disturbances and to assess skeletal calcification responses

more precisely.

Moreover, there were multiple sources of sediment load in Sumiyo Bay (silk fabric, farming, forestry, construction, and quarries). The pathway of these anthropogenic sediments to Sumiyo Bay are the same, which is transported by rivers towards the ocean with freshwater/flood plumes, regardless of the sources are different. Corals record the chemical composition of the habitat marine environment while growing by adding calcium carbonate ($CaCO_3$) skeletons. Because the pathway of anthropogenic sediments to Sumiyo Bay are the same, it is difficult to completely isolate each source of sediment (i.e., each industry) using our skeletal geochemical records (skeletal Sr/Ca, Ba/Ca, Mg/Ca, $\delta^{18}O$, $\delta^{18}O_{sw}$, and $\delta^{13}C$). There were a few previous studies that have detected the signals of the anthropogenic factors on the other skeletal geochemical records, for example, skeletal Cd/Ca[50-52], Mn/Ca[53-54], Pb/Ca[52,54-55], Y/Ca[56], V/Ca[52], Zn/Ca[56], and $\delta^{15}N$[57-58]. Because these proxies also have a complexity in the natural controlling factors (e.g., upwelling, ENSO, river discharge, and biological activity)[59], the additional study cases and combined discussions might be needed in order to completely isolate each source of natural/anthropogenic factor.

References (Supplementary Information)

1. Sumiyo Village History Editorial Committee. *Wakya shima nu ayumi* (*a history of Sumiyo village*) Chapter 2, 107–315 (Sumiyo Village History Editorial Committee, 2005. *In Japanese*).

2. Amami City. Official report on the October 2010 heavy rainfall disaster in Amami. (Amami City, 2013. *In Japanese*).

3. Kagoshima University. Reports on comprehensive scientific research for the 2010 heavy rainfall disaster in Amami (Research and Education Center for Natural Hazards, Kagoshima University, 2012. *In Japanese*).

4. Juillet-Leclerc, A. & Schmidt, G. A calibration of the oxygen isotope paleothermometer of coral aragonite from *Porites*. *Geophys. Res. Lett*. **28**, 4135–4138 (2001).

5. Cahyarini, S. Y., Pfeiffer, M., Timm, O., Dullo, W. C. & Schönberg, D. G. Reconstructing seawater $\delta^{18}O$ from paired coral $\delta^{18}O$ and Sr/Ca ratios: Methods, error analysis and problems, with examples from Tahiti (French Polynesia) and Timor (Indonesia). *Geochim. Cosmochim. Acta* **72**, 2841–2853 (2008).

6. LaVigne, M., Grottoli, A. G., Palardy, J. E. & Sherrell, R. M. Multi-colony calibrations of coral Ba/Ca with a contemporaneous in situ seawater barium record. *Geochim. Cosmochim. Acta* **179**, 203–216 (2016).

7. Chaudhuri, P. & Marron, J. S. SiZer for exploration of structures in curves. *J. Am. Stat. Assoc.* **94**(447), 807–823 (1999).

8. R Core Team. R: A language and environment for statistical computing. R Foundation for Statistical Computing, Vienna, Austria. https://www.R-project.org/ (2018).

9. Sonderegger, D. SiZer: SiZer: Significant Zero Crossings. R package version 0.1-4. https://CRAN.R-project.org/package=SiZer (2012).

10. Corrège, T. Sea surface temperature and salinity reconstruction from coral geochemical tracers. *Palaeogeogr. Palaeoclimatol. Palaeoeco*l. **232**(2–4), 408–428 (2006).

11. Mitsuguchi, T., Matsumoto, E., Abe, O., Uchida, T. & Isdale, P. J. Mg/Ca thermometry in coral skeletons. *Science*

274, 961 (1996).

12. Yu, T. L. *et al*. Geochemical effects of biomass burning and land degradation on Lanyu Islet, Taiwan. *Limnol. Oceanogr.* **60**, 411–418 (2015).

13. Esser, B. K. & Volpe, A. M. At-sea high-resolution chemical mapping: extreme barium depletion in North Pacific surface water. *Mar. Chem.* **79**, 67–79 (2002).

14. Fallon, S. J., McCulloch, M. T., van Woesik, R. & Sinclair, D. J. Corals at their latitudinal limits: laser ablation trace element systematics in Porites from Shirigai Bay, Japan. *Earth Planet. Sci. Lett.* **172**, 221–238 (1999).

15. Sowa, K., Watanabe, T., Kan, H. & Yamano, H. Influence of land development on Holocene *Porites* coral calcification at Nagura Bay, Ishigaki Island, Japan. *PLoS ONE* **9**, e88790; https://doi.org/10.1371/journal.pone.0088790 (2014).

16. Beck, J. W., Edwards, R. L., Ito, E., Taylor, F. W., Recy, J., Rougerie, F., Joannot, P. and Henin, C. Sea-surface temperature from coral skeletal strontium/calcium ratios. *Science* **257,** 644–647 (1992).

17. McCulloch, M. T., Gagan, M. K., Mortimer, G. E., Chivas, A. R. & Isdale, P. J. A high-resolution Sr/Ca and $\delta^{18}O$ coral record from the Great Barrier Reef, Australia, and the 1982-1983 El Nino. *Geochim. Cosmochim, Acta* **58**, 2747–2754 (1994).

18. Cahyarini, S. Y. et al. Twentieth century sea surface temperature and salinity variations at Timor inferred from paired coral $\delta^{18}O$ and Sr/Ca measurements. *J. Geophys. Res. Oceans* **119**, 4593–4604 (2014).

19. Wei, G., Sun, M., Li, X. & Nie, B. Mg/Ca, Sr/Ca and U/Ca ratios of a porites coral from Sanya Bay, Hainan Island, South China Sea and their relationships to sea surface temperature. *Palaeogeogr. Palaeoclimatol. Palaeoecol.* **162**, 59–74 (2000).

20. Watanabe, T., Winter, A. & Oba, T. Seasonal changes in sea surface temperature and salinity during the Little Ice Age in the Caribbean Sea deduced from Mg/Ca and $^{18}O/^{16}O$ ratios in corals. *Mar. Geol.* **173**, 21–35 (2001).

21. Dunbar, R. B. & Wellington, G. M. Stable isotopes in a branching coral monitor seasonal temperature variation.

Nature **293**, 453–455 (1981).

22. McConnaughey, T. A. ^{13}C and ^{18}O isotopic disequilibrium in biological carbonates: I. Patterns. *Geochim. Cosmochim. Acta* **53**, 151–162 (1989a).

23. Gagan, M. K., Chivas, A. R. & Isdale, P. J. High-resolution isotopic records from corals using ocean temperature and mass-spawning chronometers. *Earth Planet. Sci. Lett.* **121**, 549–558 (1994).

24. Gagan *et al.* Temperature and surface-ocean water balance of the mid-Holocene tropical western Pacific. *Science*, **279**(5353), 1014–1018 (1998).

25. Ren, L., Linsley, B. K., Wellington, G. M., Schrag, D. P. & Hoegh-Guldberg, O. Deconvolving the δ^{18}O seawater component from subseasonal coral δ^{18}O and Sr/Ca at Rarotonga in the southwestern subtropical Pacific for the period 1726 to 1997. *Geochim. Cosmochim. Acta* **67**(9), 1609–1621 (2003).

26. Weber, J. N., Deines, P. Weber, P. H. & Baker, P. A. Depth related changes in the ^{13}C/^{12}C ratio of skeletal carbonate deposited by the Caribbean reef-frame building coral *Montastrea annularis*: further implications of a model for stable isotope fractionation by scleractinian corals. *Geochim. Cosmochim. Acta* **40**, 31–39 (1976).

27. Grottoli, A. G. & Wellington, G. M. Effect of light and zooplankton on skeletal δ^{13}C values in the eastern Pacific corals *Pavona clavus* and *Pavona gigantea*. *Coral Reefs* **18**(1), 29–41 (1999).

28. Heikoop *et al*. Separation of kinetic and metabolic isotope effects in carbon-13 records preserved in reef coral skeletons. *Geochim. Cosmochim. Acta* **64**(6), 975–987 (2000).

29. Rosenfeld, M., Yam, R., Shemesh, A. & Loya, Y. Implication of water depth on stable isotope composition and skeletal density banding patterns in a Porites lutea colony: results from a long-term translocation experiment. *Coral Reefs* **22**(4), 337–345 (2003).

30. Nozaki Y., Rye D. M., Turekian K. K. & Dodge R. E. A 200 year record of carbon-13 and carbon-14 variations in a Bermuda coral. *Geophys. Res. Lett.* **5**(10), 825–828 (1978).

31. Swart, P. K. *et al*. The ^{13}C Suess effect in scleractinian corals mirror changes in the anthropogenic CO_2 inventory of the surface oceans. *Geophys. Res. Lett*. **37**(5), L05604; https://doi.org/10.1029/2009GL041397 (2010).

32. Felis, T., Pätzold, J., Loya, Y. & Wefer, G. Vertical water mass mixing and plankton blooms recorded in skeletal stable carbon isotopes of a Red Sea coral. *J. Geophys. Res*. **103**, 30–731 (1998).

33. Swart, P. K. Carbon and oxygen isotope fractionation in scleractinian corals: a review. *Earth-Sci. Rev*. **19**(1), 51–80 (1983).

34. McConnaughey, T. A. ^{13}C and ^{18}O isotopic disequilibrium in biological carbonates: II. In vitrosimulations of kinetic isotope effects. *Geochim. Cosmochim. Acta* **53**, 163–171 (1989b).

35. McConnaughey, T. A., Burdett, J., Whelan, J. F. & Paull, C. K. Carbon isotopes in biological carbonates: respiration and photosynthesis. *Geochim. Cosmochim. Acta* **61**(3), 611–622 (1997).

36. Lea, D. W., Shen, G. T. & Boyle, E. A. Coralline barium records temporal variability in equatorial Pacific upwelling. *Nature* **340**, 373–376 (1989).

37. Shen, G. T. *et al*. Surface ocean variability at Galapagos from 1936–1982: calibration of geochemical tracers in corals. *Paleoceanography* **7**, 563–588 (1992).

38. Tudhope, A. W., Lea, D. W., Shimmield, G. B., Chilcott, C. P. & Head, S. Monsoon climate and Arabian Sea coastal upwelling recorded in massive corals from southern Oman. *Palaios* **11**(4), 347–361 (1996).

39. Chen, C. C., Hsu, S. C., Jan, S. & Gong, G. C. Episodic events imposed on the seasonal nutrient dynamics of an upwelling system off northeastern Taiwan. *J. Mar. Syst.* **141**, 128–135 (2015).

40. Kawakubo, Y., Alibert, C. & Yokoyama, Y. A reconstruction of subtropical western North Pacific SST variability back to 1578, based on a *Porites* Coral Sr/Ca record from the northern Ryukyus, Japan. *Paleoceanography* **32**(12), 1352–1370 (2017).

41. Yuan, Y. *et al*. The Kuroshio east of Taiwan and in the East China Sea and the currents east of Ryukyu Islands during early summer of 1996. *J. Oceanogr.* **54**(3), 217–226 (1998).

42. Ichikawa, H., Nakamura, H., Nishina, A. & Higashi, M. Variability of northeastward current southeast of northern Ryukyu Islands. *J. Oceanogr.* **60**, 351–363 (2004).

43. Thoppil, P. G., Metzger, E. J., Hurlburt, H. E., Smedstad, O. M. & Ichikawa, H. The current system east of the Ryukyu Islands as revealed by a global ocean reanalysis. *Prog. Oceanogr.* **141**, 239–258 (2016).

44. Horibe, Y. & Ogura, N. Deuterium content as a parameter of water mass in the ocean. *J. Geophys. Res*. **73**(4), 1239–1249 (1968).

45. Sinclair, D. J. Non-river flood barium signals in the skeletons of corals from coastal Queensland, Australia. *Earth Planet. Sci. Lett*. **237**, 354–369 (2005).

46. Chen, T., Yu, K., Li, S., Chen, T. & Shi, Q. Anomalous Ba/Ca signals associated with low temperature stresses in *Porites* corals from Daya Bay, northern South China Sea. *J. Environ. Sci*. **23**, 1452–1459 (2011).

47. Lewis, S. E., Shields, G. A., Kamber, B. S. & Lough, J. M. A multi-trace element coral record of land-use changes in the Burdekin River catchment, NE Australia. *Palaeogeogr. Palaeoclimatol. Palaeoecol*. **246**, 471–487 (2007).

48. Gillikin, D. P. *et al*. Barium uptake into the shells of the common mussel (*Mytilus edulis*) and the potential for estuarine paleo-chemistry reconstruction. *Geochim. Cosmochim. Acta* **70**, 395–407 (2006).

49. Nagtegaal, R. *et al*. Spectral luminescence and geochemistry of coral aragonite: effects of whole-core treatment. *Chem. Geol.* **318**, 6–15 (2012).

50. Shen, G. T., Boyle, E. A. & Lea, D. W. Cadmium in corals as a tracer of historical upwelling and industrial fallout. *Nature* **328**(6133), 794–796 (1987).

51. Shen, G.T. & Boyle, E.A. Determination of lead, cadmium and other trace metals in annually-banded corals. *Chem. Geol.* **67**(1), 47–62 (1988).

52. Tanaka, K. *et al*. Metal contents of *Porites* corals from Khang Khao Island, Gulf of Thailand: Anthropogenic input of river runoff into a coral reef from urbanized areas, Bangkok. *Appl. Geochem.* **37**, 79–86 (2013).

53. Moyer, R. P., Grottoli, A. G. & Olesik, J. W. A multiproxy record of terrestrial inputs to the coastal ocean using

minor and trace elements (Ba/Ca, Mn/Ca, Y/Ca) and carbon isotopes ($\delta^{13}C$, $\Delta^{14}C$) in a nearshore coral from Puerto Rico. *Paleoceanography* **27,** PA3205; https://doi.org/10.1029/2011PA002249 (2012).

54. Inoue, M. *et al*. Evaluation of Mn and Fe in coral skeletons (*Porites* spp.) as proxies for sediment loading and reconstruction of 50 yrs of land use on Ishigaki Island, Japan. *Coral Reefs* **33**(2), 363–373 (2014).

55. Shen, G.T. & Boyle, E.A. Lead in corals: reconstruction of historical industrial fluxes to the surface ocean. *Earth Planet. Sci. Lett.* **82**(3), 289–304 (1987).

56. Fallon, S. J., White, J. C. & McCulloch, M. T. *Porites* corals as recorders of mining and environmental impacts: Misima Island, Papua New Guinea. *Geochim. Cosmochim. Acta* **66**(1), 45–62 (2002).

57. Yamazaki, A., Watanabe, T., Tsunogai, U., Hasegawa, H. & Yamano, H. The coral $\delta^{15}N$ record of terrestrial nitrate loading varies with river catchment land use. *Coral Reefs* **34**(1), 353–362 (2015).

58. Murray, J., Prouty, N. G., Peek, S. & Paytan, A. Coral Skeleton $\delta^{15}N$ as a Tracer of Historic Nutrient Loading to a Coral Reef in Maui, Hawaii. *Sci. Rep.* **9**(1), 5579; https://doi.org/10.1038/s41598-019-42013-3 (2019).

59. Saha, N., Webb, G. E. & Zhao, J. X. Coral skeletal geochemistry as a monitor of inshore water quality. *Sci. Total Environ.* **566**, 652–684 (2016).
